# Autism related traits and anxiety in the general population are linked through intolerance of uncertainty and affect labeling

**DOI:** 10.1038/s41598-026-47237-8

**Published:** 2026-05-12

**Authors:** Akitaka Fujii, Masahiro Hirai

**Affiliations:** 1 Department of Cognitive and Psychological Sciences, Graduate School of Informatics, Nagoya University, Nagoya, Japan; 2 Department of Pediatrics, Jichi Medical University, Shimotsuke, Japan

**Keywords:** Anxiety, Autistic traits, Intolerance of uncertainty, Affect labeling, Neuroscience, Psychology, Psychology

## Abstract

Anxiety is prevalent in autism spectrum disorder and linked to intolerance of uncertainty (IU). As a cognitive strategy, affect labeling (AL) reduces distress by structuring ambiguous sensations. However, autistic individuals frequently exhibit AL deficits, raising a critical dilemma: a strategy alleviating uncertainty-driven anxiety is inherently difficult to access. It is unclear whether IU paradoxically motivates AL use despite these deficits. In this cross-sectional study, 505 adults completed measures of autistic traits, IU, AL, and anxiety. We tested two serial mediation models: the Cognitive-Motivational Model (CMM; IU motivates AL) and Emotion Regulation deficit Model (ERM; AL deficits increase IU). While both fit well, CMM was selected based on theoretical consistency. This was consistent with established risk pathways where autistic traits relate to anxiety via higher IU and lower AL. Crucially, a novel adaptive pathway emerged: higher autistic traits were associated with higher IU, which were linked to higher AL and lower anxiety. While these pathways represent theoretical assumptions rather than proven causality, the findings suggest a dual role of IU as risk factor and motivational driver. This may point to a conflict in individuals with high autistic traits: struggling with AL deficits yet motivated to use AL to cope with uncertainty.

**Supplementary Information:**

The online version contains supplementary material available at 10.1038/s41598-026-47237-8.

## Introduction

Autism spectrum disorder (ASD) is a neurodevelopmental disorder with a reported prevalence of approximately 1 in 36 individuals^1^. Core features include persistent difficulties in social communication and social interaction, accompanied by restricted and repetitive behaviors (RRBs)^2^. Although these core features significantly affect an individual’s daily life^3–6^, the presence of co-occurring psychiatric conditions, particularly anxiety disorders, is widely recognized. Meta-analytic findings indicate that approximately 40% of autistic individuals meet the diagnostic criteria for at least one anxiety disorder at some point in their lives^7,8^, far exceeding the prevalence in the general population^9^.

Recent studies have identified “intolerance of uncertainty” (IU)^10^ as a key concept related to anxiety among autistic individuals^11–13^. IU is conceptualized as a temperamental trait characterized by a tendency to react negatively—at the cognitive, emotional, and behavioral levels—to uncertain situations or unexpected events^10,14^. Originally developed as a key component of generalized anxiety disorder^10,15^, the IU model was extended to ASD, offering a framework for understanding its association with anxiety^16,17^. Autistic individuals exhibited higher IU levels than neurotypical groups^16–18^. Supporting the role of IU in anxiety, a meta-analysis reported a significant positive association between these factors in autistic individuals^12^, and causal modeling studies consistently support the mediating role of IU^16,18–22^.

Two primary strategies have been reported to cope with elevated IU in autistic individuals. The first is a behavioral strategy called RRBs. Semi-structured interviews suggest that RRBs enhance predictability and provide a sense of control in the external environment^23–25^. Additionally, meta-analytic evidence shows a positive association between RRBs, IU, and anxiety^11^. This association can be interpreted not as RRBs causing anxiety but as an attempt to cope with high underlying anxiety^26^. This view aligns with the theoretical proposition that RRBs function as an attempt to reduce uncertainty and unpredictability^11,13,16,27^. The second is a cognitive strategy: dichotomous thinking^20,28^, which can be viewed as an attempt to reduce anxiety by simplifying complex and ambiguous information into binary categories, thus making the world appear simple and predictable^29^. However, Shi and Hirai^20^ argued that although anxiety and dichotomous thinking are associated with IU, they may constitute independent constructs. Thus, dichotomous thinking may not directly reduce anxiety; rather, both dichotomous thinking and anxiety may stem from IU. Taken together, behavioral and cognitive evidence indicates that these strategies are likely driven by the motivation to cope with the elevated IU.

This motivation may originate from difficulty in directly addressing the internal world, which arises from atypical sensory function and alexithymia, the latter of which is defined as difficulty in identifying and describing one’s own emotions^30^. Both traits are often observed in autistic individuals^31,32^. Furthermore, previous research suggests that these characteristics can heighten IU, leading to internalizing problems such as anxiety^13,19,21,22,33,34^. Thus, owing to the difficulty in managing their internal world directly, autistic individuals may employ compensatory strategies, such as RRBs and dichotomous thinking, rather than direct internal strategies.

Affect labeling (AL), defined as the act of putting feelings into words^35^, is a direct cognitive strategy that can reduce anxiety. For example, AL is associated with lower anxiety^36^, reduced physiological arousal during stress-inducing tasks^37^, and suppressed avoidance behavior in phobia^38^. These findings suggest that AL may contribute to anxiety regulation. Regarding underlying mechanisms, AL has been shown to reduce activity in the amygdala, which responds to uncertainty^39,40^. Satpute and Lindquist^41^ suggested that the reduction in amygdala activity from AL may stem from a reduction in uncertainty about affect, rather than the suppression of discrete emotions. Similarly, Torre and Lieberman^35^ also proposed the “reduction of uncertainty” as a core mechanism of AL.

As AL in autistic individuals is frequently impaired due to co-occurring alexithymia^32,42^, this raises a critical dilemma: the very strategy that could alleviate uncertainty-driven anxiety is inherently difficult to use. Crucially, however, theoretical models posit that the intense “fear of the unknown” inherent in IU drives a striving for agency to control unpredictable experiences^14^. Moreover, high IU is theoretically linked to a need for cognitive closure, characterized by an urgency to resolve ambiguity^43,44^. This drive may motivate individuals to employ AL, in an active attempt to “seize” a label for their undifferentiated sensations, essentially forcing a resolution to the distress of internal uncertainty, despite the inherent difficulty. Consequently, this motivation may compel individuals to utilize AL to structure ambiguous internal sensations, effectively overriding the cognitive challenges posed by alexithymia. Consistent with this theoretical possibility, language-based internal strategies may still be effective for emotion regulation in autistic individuals. Approaches ranging from emotion education programs^45–47^ to inner speech—defined as silent thoughts and dialogues occurring in one’s mind^48^—and private speech^49,50^ have been linked to positive outcomes such as successful emotion regulation. These findings suggest that despite the inherent challenges, the verbalization of internal states, a core component of AL, remains a viable and potentially powerful strategy for managing internal uncertainty in ASD.

Although both IU and AL are associated with anxiety and uncertainty, their causal relationship remains unclear. It is unknown whether IU motivates the use of AL as an adaptive strategy or whether difficulties in AL contribute to elevated IU. To address this theoretical gap in light of previous studies, we propose two competing hypotheses: the Emotion Regulation deficit Model (ERM) and Cognitive-Motivational Model (CMM).

The ERM posits that difficulties in AL may be associated with anxiety through the development of IU. AL is a potent internal strategy that reduces distress by providing structures for undifferentiated sensations^35,51^. However, AL is commonly impaired in autistic individuals due to co-occurring alexithymia^32,42^. Therefore, chronic exposure to “internal uncertainty” caused by impaired AL is believed to elevate IU. This view aligns with models identifying alexithymic traits as a source of IU, which subsequently contributes to internalizing problems such as anxiety^13,33,34^. Accordingly, this model predicts the following serial mediation pathway: High Autistic Traits → Low AL→ High IU → High Anxiety. In this pathway, a more fundamental mechanism is assumed, in which difficulties in AL increase IU.

Alternatively, the CMM posits that IU is a more fundamental trait that influences the motivation to use AL. This model suggests that high IU may paradoxically promote anxiety reduction. Individuals with high IU are highly motivated to make uncertain and ambiguous situations clear and predictable because of their strong aversion to uncertainty^14,43^. This motivation often promotes compensatory strategies such as RRBs in autistic individuals^23–25^, because directly addressing internal sensation and affect is challenging. However, affect itself is inherently ambiguous and elusive^51^. Consequently, high IU may promote the active use of AL as a direct coping strategy to clarify and control one’s internal state, functioning in a way similar to RRBs or dichotomous thinking. Therefore, this model predicts a serial mediation pathway opposite to the ERM: High Autistic Traits → High IU → High AL → Low Anxiety. This model suggests a more complex role for IU, proposing that it can be associated with maladaptive outcomes as well as, under certain conditions, drive adaptive cognitive strategies such as AL. However, this raises a critical dilemma given the inherent difficulty in AL due to co-occurring alexithymia^32,42^.

Based on these theoretical perspectives and the dimensional view of autistic traits in the general population, we first sought to examine these mechanisms in a non-clinical sample. Therefore, the present study aims to examine the mediating roles of IU and AL in the relationship between autistic traits and anxiety by testing two competing serial mediation models: the ERM (positing that AL deficits increase IU) versus the CMM (positing that IU motivates AL) in a general Japanese adult population (aged 20–39 years). We utilized four self-report measures: the Autism-Spectrum Quotient (AQ)^52^, Intolerance of Uncertainty Scale (IUS-12)^53^, Affect Labeling Questionnaire (ALQ)^36^, and State-Trait Anxiety Inventory (STAI)^54^ to assess autistic traits, IU, AL, and anxiety, respectively.

## Results

Internal consistency (Cronbach’s α) was reasonable for the IUS-12, ALQ, and STAI (0.88, 0.86, and 0.92, respectively), while somewhat lower for AQ (0.65). Table 1 presents descriptive statistics and Pearson’s correlations for all variables.

**Table 1:** Descriptive statistics and Pearson’s correlation coefficients among the observed variables.

	Measure	M (SD)	1	2	3	4	5	6	7	8	9	10	11
1	AQ Social skill	6.02 (2.32)											
2	AQ Attention switching	5.50 (1.82)	0.39***										
3	AQ Attention-to-detail	4.76 (1.96)	− 0.14**	0.06									
4	AQ Communication	4.67 (2.05)	0.36***	0.43***	− 0.01								
5	AQ Imagination	4.56 (1.75)	0.23***	0.17***	− 0.11*	0.37***							
6	IU Prospective anxiety	20.79 (5.08)	0.14***	0.23***	0.04	0.21***	0.07						
7	IU Inhibitory anxiety	14.33 (4.12)	0.22***	0.32***	0.03	0.33***	0.19***	0.68***					
8	ALQ Affective awareness	9.14 (2.28)	− 0.12*	− 0.14**	0.07	− 0.25***	− 0.23***	0.24***	0.07				
9	ALQ Affect labeling tendency	11.88 (3.11)	− 0.12**	− 0.14**	0.02	− 0.17***	− 0.21***	0.21***	0.05	0.58***			
10	ALQ Affect labeling capacity	8.65 (2.30)	− 0.27***	− 0.24***	0.07	− 0.30***	− 0.22***	0.13*	− 0.04	0.60***	0.55***		
11	STAI State anxiety	47.14 (10.21)	0.25***	0.27***	− 0.01	0.36***	0.27***	0.19***	0.35***	− 0.31***	− 0.27***	− 0.33***	
12	STAI Trait anxiety	49.88 (9.69)	0.37***	0.38***	− 0.02	0.36***	0.23***	0.42***	0.50***	− 0.16***	− 0.08*	− 0.29***	0.69***

**Fig. 1:**
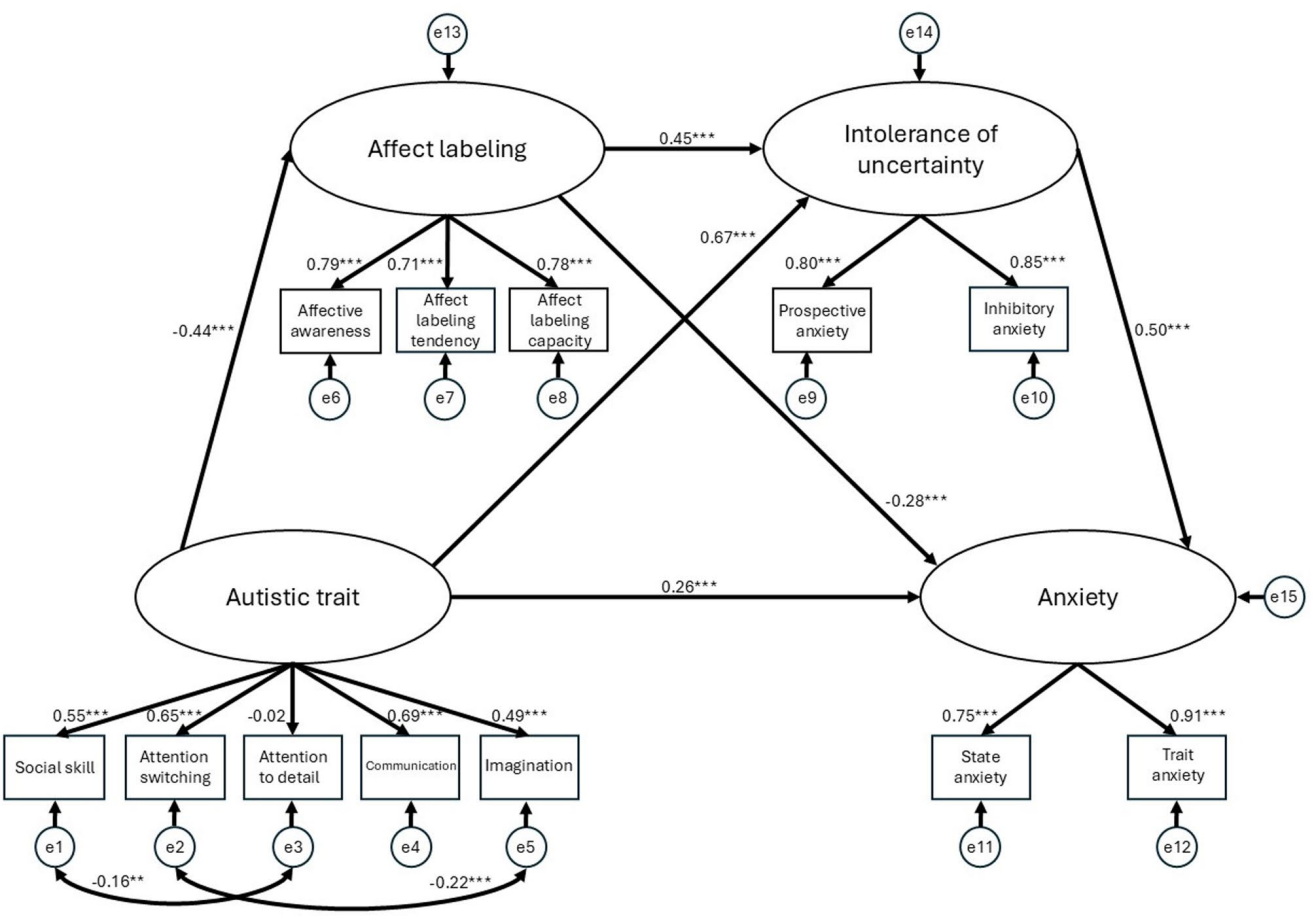
ERM: The best-fitting model. ERM: Emotion Regulation deficit Model. Ovals represent latent variables, while rectangles represent observed indicators (subscales). The value on each path indicates the standardized coefficients (β) as direct effects. Significance levels are denoted as: **p < 0.01, and ***p < 0.001. Small circles (e1–e12) denote measurement errors for the observed subscales, whereas the other small circles (e13–e15) represent residual errors for the latent variables. Double-headed arrows between error terms (e.g., e1 ↔ e3 and e2 ↔ e5) represent allowed correlations between errors, based on modification indices to account for shared variance between specific subscales of autistic traits. The positive path from affect labeling to intolerance of uncertainty (β = 0.45) is inconsistent with our a priori alexithymia-based expectations, where lower affect labeling, interpreted as a manifestation of alexithymia, is typically linked to higher intolerance of uncertainty.

**Fig. 2:**
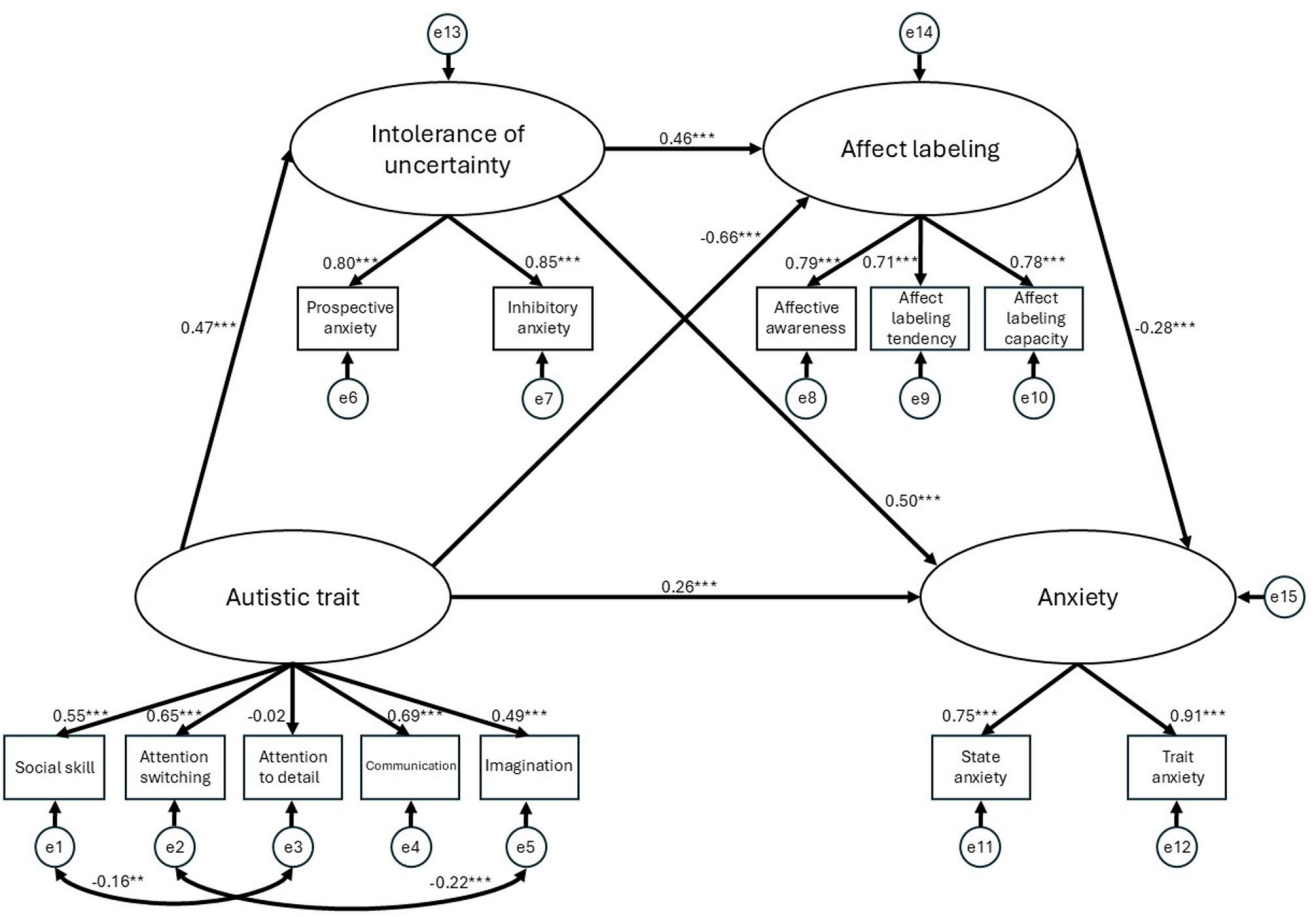
CMM: The best-fitting model. CMM: Cognitive-Motivational Model. The CMM was selected based on our a priori theoretical hypothesis, although it achieved statistically identical global fit indices to the ERM (Emotion Regulation deficit Model). Ovals represent latent variables, while rectangles represent the observed indicators (subscales). The value on each path indicates the standardized coefficients (β) as direct effects. Significance levels are denoted as: **p < 0.01, and ***p < 0.001. Small circles (e1–e12) denote measurement errors for the observed subscales, while the circles (e13–e15) represent residual errors for the latent variables. Double-headed arrows between error terms (e.g., e1 ↔ e3 and e2 ↔ e5) represent correlations between errors allowed based on modification indices, to account for shared variance between the specific subscales of the autistic traits. The positive path from intolerance of uncertainty to affect labeling (β = 0.46) is consistent with our a priori hypothesis that high intolerance of uncertainty may motivate the active use of affect labeling as a coping strategy in individuals with high autistic traits.

*M*: mean; *SD*: standard deviation; AQ: Autism-Spectrum Quotient; IU: Intolerance of Uncertainty; ALQ: Affect Labeling Questionnaire; STAI: State-Trait Anxiety Inventory. All reported subscales served as the observed indicators for the latent variables within the structural equation modeling (see Figs. 1 and 2). *p < 0.05, **p < 0.01, and ***p < 0.001.

### Emotion regulation deficit model (ERM)

The ERM demonstrated acceptable fit to the data (χ²(46) = 184.13, *p* < 0.001; χ²/df = 4.00, goodness-of-fit index [GFI] = 0.94, adjusted goodness-of-fit index [AGFI] = 0.90, comparative fit index [CFI] = 0.93, root mean square error of approximation [RMSEA] = 0.077, standardized root mean squared residual [SRMR] = 0.059, Akaike information criterion [AIC] = 248.13). Figure 1 illustrates the best-fitting model.

Within this model, autistic traits demonstrated wide-ranging influences on direct effects. Higher autistic traits were associated with higher IU (standardized direct effect *β* = 0.67, direct effect *b* = 1.99, 95% confidence interval [*CI*] [1.47, 2.59], standard error [*SE*] = 0.24, *p* < 0.001), higher anxiety (*β* = 0.26, *b* = 1.98, 95% *CI* [0.89, 3.26], *SE* = 0.59, *p* < 0.001), and lower AL (*β* = − 0.44, *b* = − 0.83, 95% *CI* [− 1.14, − 0.58], *SE* = 0.12, *p* < 0.001). Turning to the mediators, both IU and AL were significantly associated with anxiety, but in opposite directions. As hypothesized, higher IU was associated with higher anxiety (*β* = 0.50, *b* = 1.27, 95% *CI* [0.87, 1.72], *SE* = 0.17, *p* < 0.001), whereas higher AL was associated with lower anxiety (*β* = − 0.28, *b* = − 1.12, 95% *CI* [− 1.85, − 0.50], *SE* = 0.26, *p* < 0.001). However, contrary to the model’s underlying hypothesis, a positive association emerged between the mediators themselves: higher AL was associated with higher IU (*β* = 0.45, *b* = 0.71, 95% *CI* [0.44, 0.97], *SE* = 0.11, *p* < 0.001).

Regarding the indirect effects (Table 2), our results first replicated the well-established pathway through which autistic traits were positively associated with anxiety, mediated by IU (indirect effect *b* = 2.52, 95% CI [1.75, 3.73], *p* < 0.001; standardized indirect effect *β* = 0.34). In addition, our study identified a second significant pathway, consistent with our hypothesis that AL mediates this relationship (*b* = 0.93, 95% CI [0.42, 1.64], *p* = 0.001; *β* = 0.12). This reflected a pattern where higher autistic traits were associated with lower AL, which in turn was associated with higher anxiety. However, the analysis further revealed several complex indirect effects representing inconsistent mediation (i.e., suppression effects). Contrary to our main hypothesis, higher AL was positively associated with anxiety via IU (*b* = 0.90, 95% CI [0.48, 1.47], *p* = 0.001; *β* = 0.23), as higher AL was related to higher IU, which was then linked to higher levels of anxiety. Furthermore, autistic traits were negatively associated with IU, mediated by AL (*b* = − 0.59, 95% CI [− 0.96, − 0.35], *p* = 0.001; *β* = − 0.20), and negatively associated with anxiety, mediated by both AL and IU (*b* = − 0.75, 95% CI [− 1.36, − 0.38], *p* < 0.001; *β* = − 0.10). These pathways reflected a statistical pattern in which higher autistic traits were associated with lower AL, which in turn was linked to lower IU and ultimately associated with lower anxiety.

**Table 2:** Indirect effects and 95% confidence intervals for the ERM.

Model pathways	Estimated	95% CI	
		Lower	Upper
Autistic trait → Affect labeling → Intolerance of uncertainty	− 0.59**	− 0.96	− 0.35
Autistic trait → Affect labeling → Anxiety	0.93**	0.42	1.64
Autistic trait → Intolerance of uncertainty → Anxiety	2.52***	1.75	3.73
Affect labeling → Intolerance of uncertainty → Anxiety	0.90**	0.48	1.47
Autistic trait → Affect labeling → Intolerance of uncertainty → Anxiety	− 0.75***	− 1.36	− 0.38

ERM: Emotion Regulation deficit Model. The pathways describe the specific indirect effects estimated within the serial mediation framework. The estimates are unstandardized coefficients (b) with 95% bootstrap confidence intervals (CI) derived from 2,000 resamples. An indirect effect is considered statistically significant if the 95% CI does not include zero. **p < 0.01 and ***p < 0.001. An indirect effect represents inconsistent mediation (suppression effect) when its sign is opposite to the direct effect. In this model, pathways involving both affect labeling and intolerance of uncertainty represent this pattern. This occurs due to the positive relationship observed between these variables (*β* = 0.45 in Fig. 1), which operates in the opposite direction to traditional expectations, where lower affect labeling is interpreted as a manifestation of trait-level alexithymia.

### Cognitive-motivational model (CMM)

The CMM demonstrated acceptable fit to the data (χ²(46) = 184.13, *p* < 0.001; χ²/df = 4.00, GFI = 0.94, AGFI = 0.90, CFI = 0.93, RMSEA = 0.077, SRMR = 0.059, AIC = 248.13), identical to that of the ERM. Figure 2 illustrates the best-fit model. Statistically, because the two models yielded equivalent fit indices, they could not be distinguished based solely on the data. However, the CMM was selected based on the hypothesis.

Within this model, regarding the direct effects, the directionality of all paths was consistent with that of the previous model. Higher autistic traits were associated with higher IU (*β* = 0.47, *b* = 1.40, 95% *CI* [0.94, 1.84], *SE* = 0.19, *p* < 0.001), higher anxiety (*β* = 0.26, *b* = 1.98, 95% *CI* [0.89, 3.26], *SE* = 0.59, *p* < 0.001), and lower AL (*β* = − 0.66, *b* = − 1.24, 95% *CI* [− 1.64, − 0.93], *SE* = 0.16, *p* < 0.001). Regarding the mediators, higher IU was associated with higher anxiety (*β* = 0.50, *b* = 1.27, 95% *CI* [0.87, 1.72], *SE* = 0.17, *p* < 0.001), whereas higher AL was associated with lower anxiety (*β* = − 0.28, *b* = − 1.12, 95% *CI* [− 1.85, − 0.50], *SE* = 0.26, *p* < 0.001). The core mechanism of this model was centered on the effects of mediators. Crucially, and in line with the model’s name, higher IU was associated with higher AL (*β* = 0.46, *b* = 0.29, 95% *CI* [0.16, 0.41], *SE* = 0.04, *p* < 0.001).

Regarding the indirect effects (Table 3), similar to the ERM, autistic traits were positively associated with anxiety through two mediating pathways: via AL (*b* = 1.38, 95% CI [0.63, 2.45], *p* = 0.001; *β* = 0.18) and via IU (*b* = 1.77, 95% CI [1.22, 2.65], *p* < 0.001; *β* = 0.24). Furthermore, the analysis was consistent with the model’s key theoretical pathway. Notably, the indirect effects for pathways involving both IU and AL represent inconsistent mediation. Autistic traits were positively associated with AL, mediated by IU (*b* = 0.40, 95% CI [0.25, 0.62], *p* < 0.001; *β* = 0.22). This finding is consistent with the hypothesis that a high IU may be linked to greater use of AL. Additionally, the analysis revealed two negative indirect effects of anxiety. First, higher IU scores were negatively associated with anxiety when mediated by AL (*b* = − 0.32, 95% CI [− 0.65, − 0.12], *p* = 0.001; *β* = − 0.13). This suggests a pattern where, while the direct association between IU and anxiety was positive, an indirect pathway via AL showed a negative association, thereby representing a potential coping strategy. Second, autistic traits were negatively associated with anxiety, mediated by both IU and AL (*b* = − 0.45, 95% CI [− 0.95, − 0.19], *p* < 0.001; *β* = − 0.06). This three-step pathway reflects a statistical pattern in which higher autistic traits were associated with higher IU, which, in turn, was linked to higher AL and ultimately associated with lower anxiety.

**Table 3:** Indirect effects and 95% confidence intervals for the CMM.

Model pathways	Estimated	95% CI	
		Lower	Upper
Autistic trait → Intolerance of uncertainty → Affect labeling	0.40***	0.25	0.62
Autistic trait → Affect labeling → Anxiety	1.38**	0.63	2.45
Autistic trait → Intolerance of uncertainty → Anxiety	1.77***	1.22	2.65
Intolerance of uncertainty → Affect labeling → Anxiety	− 0.32**	− 0.65	− 0.12
Autistic trait → Intolerance of uncertainty → Affect labeling → Anxiety	− 0.45***	− 0.95	− 0.19

CMM: Cognitive-Motivational Model. The pathways describe the specific indirect effects estimated within the serial mediation framework. The estimates are unstandardized coefficients (b) with 95% bootstrap confidence intervals (CI) derived from 2,000 resamples. An indirect effect is considered statistically significant if the 95% CI does not include zero. **p < 0.01 and ***p < 0.001. An indirect effect represents inconsistent mediation (suppression effect) when its sign is opposite to the direct effect. In this model, pathways involving both intolerance of uncertainty and affect labeling represent this pattern. This occurs due to the positive relationship between these variables (*β* = 0.46 in Fig. 2). This supports our a priori hypothesis that high intolerance of uncertainty may motivate the active use of affect labeling as a coping strategy in individuals with high autistic traits.

## Discussion

To the best of our knowledge, this is the first study to investigate the mediating roles of IU and AL in the relationship between autistic traits and anxiety, conceptualizing AL as an emotion-regulation strategy. We proposed and compared two competing models—the ERM and the CMM—using covariance structure analysis in a general adult population. Although both models demonstrated acceptable and statistically identical fits to the data, we focused our subsequent discussion on the CMM as the primary framework based on theoretical consistency. Crucially, identical fits reinforce the fact that cross-sectional data alone do not adjudicate the directionality of these relationships^55^. This implies that our directional paths represent theoretical assumptions rather than empirically proven relationships. Given this limitation, we applied this structural equation modeling framework to evaluate the theoretical plausibility of the hypothesized pathways, as an exploratory step toward understanding the motivational aspects of IU and AL.

The CMM supported the established “risk pathway” whereby higher autistic traits are associated with higher anxiety via greater IU ^12,16^ as well as via lower AL. While research on AL in this context has often focused on alexithymia, its role as an adaptive emotion regulation strategy has been less explored. This result is consistent with findings that alexithymia traits, particularly difficulties in identification, are associated with anxiety in ASD^56^. Consistent with Sahi et al. ^36^, higher AL was associated with lower anxiety. Together, these results suggest that, while deficits in AL are risk factors, the use of AL functions as an adaptive emotion regulation strategy^35^.

Importantly, the CMM suggested a novel “adaptive pathway” characterized by inconsistent mediation: higher autistic traits were associated with higher IU, which in turn was linked to higher AL, ultimately associated with lower anxiety. These findings suggest a potential dual function: IU functions as a primary risk factor amplifying anxiety^12,16,57^ while potentially serving as a motivational driver for engaging in adaptive emotion-regulation strategies, such as AL ^35^. Although this interpretation remains a theoretical assumption based on cross-sectional patterns, it suggests a profound conflict for individuals with higher autistic traits: trait-level difficulty with AL ^42,56^, coupled with an effortful attempt to engage in AL to cope with the distress of uncertainty. Similar to RRBs and dichotomous thinking as compensatory external strategies^11,28^, AL may represent a key direct internal strategy for managing uncertainty within an ambiguous internal world. This mechanism observed in our trait-based sample may parallel the experiences of autistic individuals, whose internal world is considered particularly uncertain, both theoretically (predictive coding in ASD)^58,59^ and subjectively^23–25^. As a coping mechanism, AL can reduce the uncertainty regarding undifferentiated and ambiguous sensations^35,41^.

Unexpectedly, the results for the ERM revealed a complex pattern that challenged its initial premises. While the model partially confirmed the established risk pathways observed in the CMM^12,16,56^, it simultaneously revealed a positive association between its proposed mediators: higher AL was associated with higher IU. This finding directly contradicts the model’s premise, which, based on prior findings that alexithymia acts as a source of IU ^33,34^, predicts that lower AL would relate to higher IU. Additionally, this positive path is theoretically inconsistent with studies indicating that high alexithymic traits are a source of internal distress and anxiety in autistic individuals^42,56^.

This outcome suggests an important distinction: alexithymia as a trait is not equivalent to AL as an emotion-regulation strategy. Whereas alexithymia is linked to deficits in emotion-processing regions (e.g., insula)^60^, AL is a top-down control mechanism (e.g., right ventrolateral prefrontal cortex)^35,39^. These distinct neural underpinnings strongly support the conceptual separation between the two. Although our study did not directly measure alexithymia, Sahi et al.^36^ reveal this distinction, demonstrating that higher AL was associated with lower alexithymia. Therefore, the CMM provides one plausible framework wherein alexithymic traits may serve as a source of IU and anxiety, while AL can be a cognitive strategy motivated by IU.

Furthermore, interpreting low AL as “cognitive avoidance”—a strategic avoidance of confronting uncertain emotional experiences—is inconsistent with the observed statistical patterns. If low AL functioned as an avoidance strategy, a negative association would be expected (i.e., low AL linked to high IU)^61^. By contrast, our data revealed a positive association between AL and IU. This finding contradicts the ERM but strongly supports the CMM’s proposition that AL functions as an active regulatory strategy driven by IU, rather than an avoidance mechanism.

For these reasons, rather than suggesting that the ERM is empirically inferior, we argue that the CMM provides a more conceptually coherent theoretical framework for explaining the mechanisms in this non-clinical sample, despite adequate model fit. The choice to prioritize the CMM is thus hypothesis-driven, aimed at exploring one plausible theoretical pathway that the data alone cannot establish. To refine the CMM framework, distinguishing between the sources of IU and strategies used to manage it is essential. Although previous studies have identified factors such as alexithymia and atypical sensations as sources of IU ^19,21,22,33,34^, none have integrated these sources with AL as a coping mechanism within a single model, highlighting the need for future studies to concurrently measure these traits and examine their correlations. Additionally, future research should simultaneously compare multiple coping strategies for elevated IU, including AL as a direct internal strategy and both RRBs and dichotomous thinking as compensatory external strategies. Examining these divergent pathways could identify factors influencing strategy selection and directly inform individualized interventions.

Although this study relied on a non-clinical sample and implications for autistic individuals remain hypothetical, our findings suggest that interventions aimed at enhancing AL skills may represent a valuable therapeutic approach. While the effectiveness of emotion education for autistic individuals has been suggested^45–47^, the underlying mechanism identified in this study that IU itself can act as a motivator for AL despite these inherent difficulties, has not been clearly conceptualized. If these trait-based mechanisms extended to clinical populations, elevated uncertainty combined with difficulty in AL may contribute to heightened frustration. This cyclical struggle offers one plausible explanation for the statistical equivalence of our competing models. While high IU motivates the use of AL to reduce uncertainty, as proposed by the CMM, the inherent difficulty in labeling ambiguous affect may simultaneously sustain or exacerbate uncertainty. This highlights why unaided AL can be frustrating and emphasizes the critical need for external support that scaffolds the verbalization of feelings. Indeed, research has begun to examine the effectiveness of interpersonal AL—having one’s feelings labeled by others^62^—which aligns with the long demonstrated importance of empathizing with another person’s experiences in counseling settings^63^.

This study has some limitations. First, its cross-sectional nature limited causal inferences. Although covariance structure analysis allowed the evaluation of theoretical pathways, these results represent hypothesized directions of influence rather than proven causality. For instance, while we positioned IU as a predictor of anxiety, it remains possible that elevated anxiety could reciprocally increase an individual’s sensitivity to uncertainty, or that this relationship is bidirectional. Moreover, the substantive meaning of the observed inconsistent mediation should be interpreted with caution. Such patterns may also arise from unmeasured confounding variables or complex statistical interdependencies. Future research employing longitudinal or experimental designs is required to elucidate these temporal dynamics and verify the proposed mechanisms.

Second, there are several challenges regarding the measurement of AL. The Japanese version of the ALQ used in this study has not been formally validated. Although, as reported in the Supplementary Material, we confirmed its factor structure and internal consistency within our sample, and the exclusion of two reverse-coded items was a data-driven step based on this single sample. Furthermore, the internal consistency of the resulting 3-item subscales was marginal (0.67 and 0.69). Consequently, the present ALQ results should be viewed as preliminary, and future studies are needed to formally establish its psychometric properties. Nevertheless, a sensitivity analysis using the full 12-item ALQ yielded results consistent with our primary findings, reinforcing the stability of the observed patterns. Moreover, considering that all constructs, including AL, were assessed through self-reporting, the results may be subject to shared method variance. Furthermore, a gap may exist between self-reported tendencies and actual emotion-regulation behavior under emotional load or stress. Future studies using experimental paradigms are necessary to bridge this gap.

Third, the internal consistency of the AQ was relatively modest (0.65). Although we employed latent variable modeling to account for the measurement error, the modest reliability may still influence the precision of the latent construct’s estimation. Accordingly, the magnitude of associations involving autistic traits should be interpreted with appropriate caution.

Fourth, the limitations regarding sample generalizability must be acknowledged. As the participants consisted of a non-clinical sample of Japanese adults, the findings primarily speak to trait-level associations, and cannot be directly generalized to individuals with a clinical diagnosis of ASD. Future investigations with clinical samples are necessary to determine whether these mechanisms operate similarly in autistic individuals, particularly when accounting for co-occurring conditions such as intellectual functioning^2^. Furthermore, potential cultural influences must be considered. While the measures used were either validated or partially validated in Japanese, constructs such as AL and IU may manifest differently across cultures, and as such the observed patterns may only reflect the specific sociocultural contexts in Japan.

Finally, while CMM’s motivational framework is consistent with the idea that high IU may sometimes drive internal coping, it remains exploratory and requires replication. We acknowledge that higher AL use in individuals with higher IU—despite inherent difficulties—could reflect a specific motivational process or, alternatively, characterize a specific subgroup (e.g., those with higher deliberation processing^64,65^ or rumination^66^. Thus, these results should be interpreted as one plausible theoretical pathway consistent with our cross-sectional data, rather than as a definitive causal mechanism.

## Methods

### Participants

A total of 532 adults in the general Japanese population (male = 272, female = 260, age range = 20–39 years, mean = 30.14 years, standard deviation [*SD*] = 5.72) were recruited from a pool of individuals registered with Cross Marketing Inc. Informed consent was obtained from all participants before they responded to the questionnaire. This study was approved by the ethics committee of the Department of Cognitive and Psychological Sciences, Graduate School of Informatics, Nagoya University (NUPSY-250623-R-01), and conducted in accordance with the Declaration of Helsinki. All methods were performed in accordance with the relevant guidelines and regulations.

Recent research has emphasized the importance of screening for careless responses^67^. Among the available approaches, the long string index analysis is widely recommended as a post hoc screening method. We calculated the long string index for each participant across all independent measures using the careless package in R ^68^ and excluded participants whose identical responses exceeded a threshold of 0.4 standard deviations above the mean^69^. This procedure resulted in the exclusion of 27 participants, yielding a final sample of 505 (male = 254, female = 251, age range = 20–39 years, mean = 30.23 years, SD = 5.70), which was used for all subsequent analyses.

To further verify the robustness of our findings, we conducted supplementary analyses including age and gender as covariates in the structural models. The model fit remained acceptable, and all primary structural pathways remained statistically significant even after controlling for these demographic variables (see Supplementary Material S1).

### Materials

#### Autism-spectrum quotient (AQ)

Autistic traits were assessed using the Japanese version of the Adult AQ ^52^. Originally developed by Baron-Cohen et al. ^70^, this 50-item self-report questionnaire was designed to measure the degree to which adults with normal intelligence exhibit traits associated with the autistic spectrum. The reliability and validity of the Japanese version have been confirmed^52^. AQ comprises five subscales: social skills (i.e., the abilities necessary to interact and engage with others), attention switching (i.e., the ability to shift attention to another object as circumstances require), attention-to-detail (i.e., the ability to notice minute details and characteristics of things), communication (i.e., the ability to communicate with others), and imagination (i.e., the ability to imagine hypothetical situations or think creatively). Items are rated on a 4-point Likert scale (strongly disagree, disagree, agree, or strongly agree). For responses associated with autistic tendencies, 1 point is assigned regardless of whether the rating chosen is “strongly.” The score is the sum of the response values, and the subscale scores range from 0 to 10.

#### State-trait anxiety inventory (STAI)

Anxiety was assessed using the Japanese version of the STAI^54^. Originally developed by Spielberger et al.^71^, this 40-item self-report questionnaire is designed to measure both state and trait anxiety. State anxiety indicates a temporary, situational state of anxiety accompanied by autonomic nervous system arousal. Trait anxiety indicates a tendency to perceive stressful situations as threatening and represents a relatively stable individual characteristic. Shimizu et al.^54^ confirmed the reliability and validity of the Japanese version. Items are rated on a 4-point Likert scale (almost never, sometimes, often, or almost always). After reverse-scoring the appropriate items, the scores for the 20-item subscales were calculated by summing the item ratings. Scores for each subscale range from 20 to 80.

#### Intolerance of Uncertainty Scale (IUS-12)

IU was assessed using the Japanese version of the short version of the IUS-12^53^. Originally developed by Carleton et al.^72^, this 12-item self-report questionnaire measures an individual’s tendency to consider uncertainty as unacceptable. The Japanese version of the IUS-12 has demonstrated adequate reliability and validity^53^. It comprises two subscales: prospective anxiety (i.e., fear and anxiety in anticipation of uncertainty; 7 items, score range: 7–35) and inhibitory anxiety (i.e., inhibition of action or experience in the face of uncertainty; 5 items, score range: 5–25). Each item is rated on a 5-point Likert scale ranging from 1 (not at all) to 5 (very much), and the total score is the sum of the response values.

#### Affect labeling questionnaire (ALQ)

AL was assessed using the ALQ^36^, a 12-item self-report questionnaire designed to measure individual differences in AL. The original version is an unpublished preprint with confirmed reliability and validity. For this study, a Japanese version was developed with the original author’s permission following a standard translation and back-translation procedure. Items are rated on a 5-point Likert scale from 1 (strongly disagree) to 5 (strongly agree). The instrument originally comprises three subscales: Affective awareness (awareness of one’s emotional states), Affect labeling tendency (the tendency to use language to process emotional states), and Affect labeling capacity (the ability to find language to describe those states).

A preliminary confirmatory factor analysis in our sample indicated that two reverse-scored items undermined the scale’s reliability. Therefore, these items were removed to minimize potential method effects^73^ and optimize internal consistency, and a 10-item version of the ALQ (Affective awareness: 3 items; Affect labeling tendency: 4 items; Affect labeling capacity: 3 items) was retained for all primary analyses. This revised scale demonstrated excellent fit (CFI = 0.98, RMSEA = 0.049) and substantially improved internal consistency: Affective awareness (*α* = 0.67), Affect labeling tendency (*α* = 0.79), Affect labeling capacity (*α* = 0.69), and total score (*α* = 0.86). The internal consistency of the 3-item subscales was considered acceptable for short scales with few items^74^. Concurrent validity was supported by significant correlations with theoretically related constructs (i.e., autistic traits and anxiety), as shown in Table 1. Importantly, the original 12-item version yielded acceptable fit indices across all hypothesized structural models, indicating that item removal was motivated by measurement quality rather than model fit considerations. The three-factor structure was retained after item removal, with all remaining items loading appropriately on their intended factors. As the goal of this study was hypothesis testing rather than scale development, we adopted the version that provided the most psychometrically stable indicators for testing the hypothesized structural model. Full psychometric results and sensitivity analyses comparing the 12-item and 10-item versions are presented in the Supplementary Materials S2 and S3.

### Data analysis

To evaluate the mediating roles of IU and AL in the relationship between autistic traits and anxiety, two competing models (ERM vs. CMM) were compared using covariance structure analysis with Analysis of Moments Structures (AMOS version 29). Descriptive statistics and correlations were analyzed using R4.5.1^75^. Model fit was assessed using the maximum likelihood (MLχ^2^) test, ratio of chi-square to degrees of freedom (χ²/df), GFI, AGFI, CFI, RMSEA, SRMR, and AIC. Following conventional criteria (e.g., CFI > 0.95, RMSEA < 0.08, and SRMR < 0.08) and recommendations by Cho et al.^76^ (fit criteria set as GFI ≥ 0.93 or SRMR ≤ 0.08 for sample sizes exceeding 100), adequate fit was determined. To test the significance of path coefficients, we employed bootstrapping with 2,000 resamples. The significance of the direct effects was assessed using p values (*p* < 0.05).

Direct effects are reported as standardized coefficients (*β*), with their *SE*, p-values, and 95% *CI* based on unstandardized coefficients (*b*). Indirect effects were reported as unstandardized coefficients (*b*) with 95% bias-corrected bootstrap *CI*; significance was determined if the CI did not contain zero. Standardized indirect effects (*β*) were included for reference.

Preliminary analyses confirmed that multicollinearity among the primary variables was not a concern (variance inflation factors < 1.30; see Supplementary Material S4).

## Supplementary Information

Below is the link to the electronic supplementary material.

Supplementary Material 1

## Data Availability

Data supporting the findings of this study are available from the corresponding author upon reasonable request.
