# Supplementary material for: Autism related traits and anxiety in the general population are linked through intolerance of uncertainty and affect labeling
Source: Sci Rep. 2026 May 12;16:13149. doi: 10.1038/s41598-026-47237-8 (PMC13168363; doi:10.1038/s41598-026-47237-8)
Supplement: Supplementary file 1 — Supplementary Material 1 [file 41598_2026_47237_MOESM1_ESM.docx]

**Autism related traits and anxiety in the general population are linked through intolerance of uncertainty and affect labeling**

Akitaka Fuji ^a*^, Masahiro Hirai^a,b*^

^a^ Department of Cognitive and Psychological Sciences, Graduate School of Informatics, Nagoya University, Japan

^b^ Department of Pediatrics, Jichi Medical University, Tochigi, Japan

**Supplementary Information**

# **Supplementary Material S1. Sensitivity analysis**: **results including age and gender as covariates**

To further verify the robustness of our findings, we conducted supplementary analyses including age and gender as covariates in the structural models. Both models (the Emotion Regulation deficit Model [ERM] and the Cognitive-Motivational Model [CMM]) demonstrated identical and acceptable fit to the data (χ²(62) = 234.81, p < .001; χ²/df = 3.79, GFI = .93, AGFI = .89, CFI = .91, RMSEA = .074, SRMR = .058, AIC = 320.81). Furthermore, all primary structural pathways remained statistically significant even after controlling for these demographic variables.

# **Supplementary Material S2. Psychometric evaluation and item selection of the Affect Labeling Questionnaire**

First, confirmatory factor analysis was conducted on the translated scale using all 12 items based on the original 3-factor model. Although the model fit indices were within an acceptable range (CFI = .93, RMSEA = .069, SRMR = .059, AIC = 15600.44), internal consistency was unacceptable for two subscales (Total: α = .79; Affective awareness: α = .44; Affect labeling tendency: α = .79; Affect labeling capacity: α = .55). An examination of the parameter estimates revealed that the standardized factor loadings for the two reverse-scored items (Q2 from Affective awareness: loading = -0.15; Q12 from Affect labeling capacity: loading = -.021) were notably low (see Supplementary Table S1). This suggests a method effect attributable to item wording (reverse coding), rather than the substantive content of the construct ^1^. Importantly, inspection of the item content confirmed that the remaining items sufficiently covered the theoretical domains of Affective awareness and Affect labeling capacity, indicating that the removal of these problematic items would not compromise content validity. Therefore, to ensure construct validity, a modified model was constructed by excluding these two items, resulting in a 10-item, 3-factor structure (Affective awareness: 3 items; Affect labeling tendency: 4 items; Affect labeling capacity: 3 items). The subsequent confirmatory factor analysis showed improved model fit indices (CFI = .98, RMSEA = .049, SRMR = .034, AIC = 12711.27). All the factor loadings were statistically significant (Supplementary Table S2). Furthermore, the internal consistency for each subscale improved to acceptable levels considering the reduced number of items (Total: α = .86; Affective awareness: α = .67; Affect labeling tendency: α = .79; Affect labeling capacity: α = .69). The internal consistency of the 3-item subscales was considered acceptable for scales with few items ^2^. Furthermore, their mean inter-item correlations values were .40 and .43, respectively; values between .15 and .50 indicate adequate internal consistency and homogeneity ^3^, especially for brief scales where α naturally yields lower values due to the small number of items.

Although the revised 3-factor model showed excellent fit, a high correlation was observed between Affective awareness and Affect labeling capacity (*r* = .88). Notably, this high correlation was also present in the original 12-item model (*r* = .87), indicating that the strong association was intrinsic to the constructs rather than an artifact of item reduction. To determine whether these constructs should be merged, we tested an alternative 2-factor model in which items from both subscales were loaded onto a single factor. A chi-square difference test was conducted to compare the hypothesized 3-factor model with an alternative 2-factor model. The results indicated that the 3-factor model provided a significantly better fit to the data (*Δχ²*(2) = 12.66, *p* = .002). Furthermore, the 3-factor model demonstrated a lower AIC value (12711.27) compared to the 2-factor model (12719.93), suggesting superior parsimony and fit. Therefore, the original 3-factor structure is used for subsequent analyses.

**Supplementary Table S1**

*Confirmatory factor analysis results for the full 12-item Japanese Version of the Affect Labeling Questionnaire.*

| Item | Affective awareness | Affect labeling tendency | Affect labeling capacity |
| --- | --- | --- | --- |
| Q1 | .638*** | - | - |
| Q2 | .-.147**…. | - | - |
| Q3 | .627*** | - | - |
| Q4 | .632*** | - | - |
| Q5 | - | .689*** | - |
| Q6 | - | .654*** | - |
| Q7 | - | .709*** | - |
| Q8 | - | .717*** | - |
| Q9 | - | - | .602*** |
| Q10 | - | - | .628*** |
| Q11 | - | - | .727*** |
| Q12 | - | - | -.021…... |
| Cronbach's α | .44…. | .79….. | .55….. |

Note: *N* = 505. Standardized factor loadings were also reported. **p* < .05, ***p* < .01, ****p* < .001. Model fit indices: χ²(51) = 173.08, p < .001; CFI = .93, TLI = .90, RMSEA = .069, SRMR = .059, AIC = 15600.44. Factor correlations: Affective awareness-Affect labeling tendency (*r* = .808), Affective awareness-Affect labeling capacity (*r* = .874), Affect labeling tendency-Affect labeling capacity (*r* = .756). The items were numbered consecutively (Q1–Q12) across the three subscales presented in Sahi et al. ^4^.

**Supplementary Table S2**

*Confirmatory factor analysis results for the 10-item Japanese version of the Affect Labeling Questionnaire.*

| Item | Affective awareness | Affect labeling tendency | Affect labeling capacity |
| --- | --- | --- | --- |
| Q1 | .644*** | - | - |
| Q3 | .621*** | - | - |
| Q4 | .640*** | - | - |
| Q5 | - | .689*** | - |
| Q6 | - | .655*** | - |
| Q7 | - | .708*** | - |
| Q8 | - | .717*** | - |
| Q9 | - | - | .603*** |
| Q10 | - | - | .631*** |
| Q11 | - | - | .726*** |
| Cronbach's α | .67…... | .79…... | .69…... |

Note: *N* = 505. Standardized factor loadings were also reported. ****p* < .001. Model fit indices: χ²(32) = 70.40, *p* < .001; CFI = .98, TLI = .97, RMSEA = .049, SRMR = .034, AIC = 12711.27. Factor correlations: Affective awareness-Affect labeling tendency (*r* = .792), Affective awareness-Affect labeling capacity (*r* = .875), and Affect labeling tendency-Affect labeling capacity (*r* = .754). The items are numbered consecutively (Q1–Q12) across the three subscales presented by Sahi et al. ^4^.

# **Supplementary Material S3. Sensitivity analysis: results using the original 12-item Affect Labeling Questionnaire**

To ensure the robustness of our findings, we tested the hypothesized structural models (ERM and CMM) using the original 12-item version of the ALQ. In both models, the results showed that the 12-item model also maintained acceptable fit to the data (χ²(46) = 204.17, *p* < .001; χ²/df = 4.44, CFI = .91, RMSEA = .083, and SRMR = .060; see Supplementary Table S3 for a full comparison of fit indices). Furthermore, the pattern of significant associations and path coefficients remained consistent with the primary model, confirming that item reduction did not alter the substantive conclusions.

**Supplementary Table S3**

*Comparison of fit indices for the hypothesized structural model (ERM and CMM) using different versions of the Affect Labeling Questionnaire.*

| Model Version | χ²/df | GFI | AGFI | CFI | RMSEA | SRMR | AIC |
| --- | --- | --- | --- | --- | --- | --- | --- |
| Primary model (10-item): ERM | 4.00 | .94. | .90. | .93. | .077. | .059. | 248.13 |
| Primary model (10-item): CMM | 4.00 | .94. | .90. | .93. | .077. | .059. | 248.13 |
| Original model (12-item): ERM | 4.44 | .93. | .88. | .91. | .083. | .060. | 268.17 |
| Original model (12-item): CMM | 4.44 | .93. | .88. | .91. | .083. | .060. | 268.17 |

Note: The primary model was estimated using the 10 selected items from the Affect Labeling Questionnaire, whereas the original model was estimated using the full 12-item scale. ERM, Emotion Regulation deficit Model; CMM, Cognitive-Motivational Model; GFI, goodness-of-fit index; AGFI, adjusted GFI; CFI, comparative fit index; RMSEA, root mean square error of approximation; SRMR, standardized root mean square residual; AIC, Akaike information criterion.

# **Supplementary Material S4: Multicollinearity check**

To ensure that the inconsistent mediation observed in the mediation models was not an artifact of multicollinearity, we calculated the variance inflation factors (VIF) for the primary predictors (AQ, IU, and AL). The VIF values were 1.30 for AQ, 1.21 for IU, and 1.19 for AL. These values are well below the conservative threshold of 2.5, confirming that the predictors are statistically distinct and that multicollinearity is not a concern in the current structural equation models.

# **References**

[1] Podsakoff, P. M., MacKenzie, S. B., Lee, J.-Y. & Podsakoff, N. P. Common method biases in behavioral research: A critical review of the literature and recommended remedies. *J. Appl. Psychol.* **88**, 879–903 (2003).

[2] Cortina, J. M. What is coefficient alpha? An examination of theory and applications. *J. Appl. Psychol.* **78**, 98–104 (1993).

[3] Clark, L. A. & Watson, D. Constructing validity: New developments in creating objective measuring instruments. *Psychol. Assess.* **31**, 1412–1427 (2019).

[4] Sahi, R. S., Moreira, J. F. G., Torre, J. B. & Lieberman, M. D. The Affect Labeling Questionnaire (ALQ): decomposing affect labeling and implications for individual differences in socio-emotional well-being. Preprint at https://doi.org/10.31234/osf.io/b8hde (2023).
